# Ethnobotanical Study of Plants Used for Human Ailments in Yilmana Densa and Quarit Districts of West Gojjam Zone, Amhara Region, Ethiopia

**DOI:** 10.1155/2021/6615666

**Published:** 2021-02-18

**Authors:** Derebe Alemneh

**Affiliations:** Department of Biology, College of Natural and Computational Sciences, Assosa University, P.O. Box 18, Assosa, Ethiopia

## Abstract

Like other populations of developing countries, most of the populations of Ethiopia depend greatly on the use of traditional herbal medicines as the primary source of health care. However, these resources have been degraded throughout the country. Therefore, recording and documenting medicinal plants are essential for the future conservation of the species. Thus, the current study is related to this issue. Three hundred ninety-five informants participated in this study. The main data collection tools were semistructured interviews, discussions, and observation. The data were analyzed using both qualitative and quantitative methods. The quantitative methods (informant consensus factor, fidelity level) were used to analyze the level of homogeneity of the data, the agreements of informants, and the healing potential of medicinal plants. The ranking exercises (preference and direct matrix ranking) were used to quantify the most preferred and multipurpose medicinal plants. A statistical test was conducted using SPSS version 20 to test knowledge differences among the people. Following the analysis, 112 species of medicinal plants with 62 families were recorded. Most of the species were herbs, of which Fabaceae was the dominant family. Blood pressure and asthma were the most commonly reported human health problems. Most of the remedies were prepared from leaves, and most of them were prepared by pounding and were applied orally. Gastrointestinal diseases had the highest informant consensus. Five medicinal plants were recorded with the highest healing potential value for malaria, asthma, tapeworm, cough, and stomach ulcer. *Verbascum sinaiticum* Benth. was the most preferred medicinal plant in treating blood pressure. *Trigonella foenum-graecum* L. had the highest use value. The analysis results showed that local people showed significant knowledge differences (*p* < 0.05). In conclusion, the indigenous people have a wealth of indigenous herbal knowledge. Therefore, to sustain the wealth of indigenous knowledge of the districts, immediate and well-designed conservation practices of medicinal plants should be conducted.

## 1. Introduction

Globally, the populations of developing countries firmly persist on using traditional medicines as their primary source of health care [1]. Populations throughout Africa use traditional medicine for the delivery of their principal health care showing that traditional medicine is a widely growing health system with economic importance [2]. Ethnobotanical studies carried out throughout Africa confirm that native plants are the main constituent of traditional African medicines [1]. According to [3], around 80% of the population of Ethiopia depends on traditional medicinal plants for their primary health care needs. In connection with this, about 95% of the traditional medicine of the country has been prepared from plants [4]. This is because of the rich indigenous knowledge on medicinal plant use [5]. Particularly, a privileged concentration of medicinal plant knowledge is found in the South and Southwestern parts of Ethiopia because of the high cultural diversity of the population of these areas [5, 6].

It is estimated that about 56,000 tons of noncultivated medicinal plants of Ethiopia were harvested in only a year [7]. This harvested amount of traditional medicinal plants was estimated to become a source of 2 billion Birr (Ethiopian currency) [8]. On one hand, this speaks of the highest economic value of medicinal plants; on the other hand, it reveals the practice of the highest consumption of medicinal plants in the country. Thus, there is the highest degradation of the vegetation resources throughout Ethiopia because of various anthropogenic factors such as overharvesting, agricultural land expansion which leads to the clearance of the vegetation in some areas, and fuelwood collection.

The Yilmana Densa and Quarit districts are some of the dry Afromontane and grassland complex regions of Ethiopia. Thus, the districts harbored a highly diversified species of woody and herbaceous plants. The diversified wealth of plants in these districts is the source of medicinal plants of the districts and the interrelated indigenous knowledge of the community. However, like other parts of the country, there is the highest dwindling of vegetation in these districts because of human-driven factors. These also led to the loss of medicinal plants and rich indigenous knowledge of the areas, and thus, the need for immediate conservation actions. Studying, recording, and documenting the medicinal plants of the areas are also one of the conservation actions. Thus, the purpose of the current study was to record, document, and analyze medicinal plants and the associated indigenous knowledge of the local community in Yilmana Densa and Quarit districts of West Gojjam Zone, Amhara Region, Ethiopia.

## 2. Methods

### 2.1. Study Area Description

#### 2.1.1. Location and Boundaries

**Figure 1:**
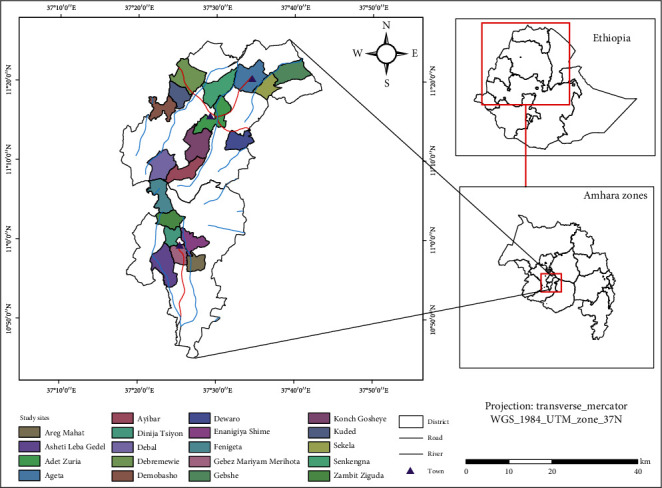
Location of the study Kebeles (smallest administrative units) in the districts of the West Gojjam Zone, Northwest Ethiopia (drawn using Arc GIS ver. 10.5).

The Yilmana Densa and Quarit districts (weredas) are two of the ten districts of the West Gojjam Zone in the Amhara Region, Ethiopia. The two districts are bordering districts selected for the purposes of increasing study area coverage and vegetation cover of the areas. The Yilmana Densa district is bordered by Bahir Dar Zuria (the district that surrounds Bahir Dar, the capital city of the Amhara Region) on the north and Abay River on the east, which separates it from the South Gondar Zone. The major town of this district is Adet (the town which is 42 kilometers far from Bahir Dar). The Quarit district is bordered on the north by Yilmana Densa and on the east by the East Gojjam Zone. The major town is Dabi/Gebez Maryam (Figure 1).

#### 2.1.2. Mainland Covers

The mainland covers in the study areas are settlements surrounded by *Eucalyptus* trees, cultivated land, grassland, woodland, and shrub/bushland. It also includes evergreens and semievergreens, small trees, and occasionally larger trees. Besides, there are a few scattered trees such as *Acacia* sp., *Cordia africana*, and *Croton macrostachyus* in the farmlands, whereas *Eucalyptus camaldulensis* is grown around the homestead [9].

#### 2.1.3. Demography

According to CSA [10], the Yilmana Densa district has a total population of 214,852, of which 107,010 are males and are 107,842 females, whereas the Quarit district has a total population of 114,771, of which 56,767 are males and 58,004 are females. The majority (98.19%) of the inhabitants of the Yilmana Densa district practiced Ethiopian Orthodox Christianity. Most of the population lives in rural areas, whereas the least number from the population inhabit urban areas. 99.9% of the district's inhabitants are composed of Amhara people, and Amharic is spoken as the first language by 99.96% [11].

#### 2.1.4. Livelihoods

The populations of the districts have several livelihoods. The first one is traditional farming using oxen, and rarely, heifers, cows, horses, and mules. Crop production is entirely rain-fed, except in a few Kebeles where small-scale water harvesting practices have been recently introduced by the Office of Agriculture and Rural Development. There is only one rainy season (summer), and it is important for the cultivation of both long- and short-cycle crops [9].

The other alternative sources of livelihood include off-farm and nonfarm activities. Nonfarm activities are practiced because of a shortage of agricultural land. Cultivated land per household is getting smaller and smaller, mainly because of high population pressure. Land and livestock productivity is declining because of natural resource depletion. The newly established houses do not have access to farmland. Thus, the communities are engaged in nonfarm activities such as pottery, metalwork, weaving, carpentry, and basket making even if their numbers are insignificant [12]. Off-farm activities (micro and small enterprises) are the second means of poverty reduction and include animal husbandry, poultry, honey production, and construction [13]. According to [14], livestock is the most valuable resource for the livelihood of the rural people. Especially, cattle are the best source of income [9]. Thus, an average annual income of 12,087,131.00 Birr has been generated from livestock production. The aggregate annual income generated from crop production is estimated at c. 19,725,688 Birr [15].

#### 2.1.5. Human Diseases

The main human diseases of the two districts are malaria, tuberculosis, lung diseases, intestinal parasites, diarrheal diseases, gastritis and duodenitis, eye diseases, skin wounds (infections), and epilepsy. The transmission of malaria increases between September and November, which are the major transmission seasons [9]. The seasons are associated with the amount of rainfall and relative humidity. The average monthly rainfall for malaria transmission was recorded to be 86.6-316.3 mm, and that of average monthly relative humidity was 50-78%. The other major disease type is tuberculosis whose consequences are serious and potentially weakens patients and their families. Like brucellosis, it can be transmitted by drinking infected milk [16]. In connection to this, a poor road network and the absence of public transport services are the other major problems that exacerbate the situation of patients that need referral services. There is also a lack of multiple health services such as delivery, injection, essential drugs, and consumable commodities [14].

### 2.2. Sample Selection

Nineteen rural Kebeles (twelve from Yilmana Densa, and seven from Quarit) were selected during a reconnaissance survey by discussing with the districts' administrators and the local people (Figure 1). A total of 395 informants (95 key informants) were selected from community members. Peer recommendations from community members aided in nominating key informants. The age of informants ranged from 20 to 81 years (139 were from 20 to 40 years old, whereas 256 were >40 years old) as stated in the method used in [17, 18].

### 2.3. Seasons of Data Collection

Data were collected during four different field visits conducted between 15 September 2016 and 30 June 2018 in different seasons over different years to collect plant specimens during the respective flowering seasons.

### 2.4. Data Collection Tools

Semistructured interviews, focus group discussions (FGD), field observation, and market surveys were used to collect data by using the method found in [17]. A semistructured interview and FGD were employed by using a checklist of questions initially prepared in English but translated into and presented in Amharic, which is the common language of the local community.

#### 2.4.1. Semistructured Interviews

The informants were interviewed individually in the local Amharic language. Semistructured interviews included questions about name, age, gender, level of education, occupation, religion, nationality, District, Kebele, and peasant association of each informant. All semistructured interviews were followed by independent walk-in-the-woods exercises to pave a way for a detailed discussion with the informants. The informants were asked about local names, habitats, use diversities, parts used, collecting households, condition of plant part used (fresh/dried), ingredients used, mode of preparation, the threats, and traditional conservation practices of medicinal plants [19]. The informants were also asked about overall use values, diseases treated, methods of remedy preparation, dosage prescriptions and routes of remedy administration, other ingredients, source of knowledge about medicinal plants, and method of indigenous knowledge transfer as stated in [20].

#### 2.4.2. Focus Group Discussion (FGD)

A focus group discussion (comprising 7 participants) per Kebele (subdivision of the district) was undertaken to gain detailed information on plant knowledge at the community level and to supplement the information collected through semistructured interviews as shown in [17].

#### 2.4.3. Field Observation

Field observations were performed with the help of local guides and some respondents of the local community for the purpose of practical identification of medicinal plants in the field.

#### 2.4.4. Market Surveys

Market surveys were conducted between 10 December 2017 and 7 May 2018. These surveys were conducted in the local marketplaces, namely Adet and Bir Gebeya (in the Yilmana Densa district) and Dabi (in the Quarit district). The purpose of the market survey was to record, document, and analyze the availability, price, and unit of measurement of traditional medicine and other useful plants following the method used in [19].

#### 2.4.5. Plant Specimen Identification

Medicinal plant specimens were collected, dried, numbered, labeled, pressed, identified, and deposited at the National Herbarium (ETH) in Addis Ababa University. Identification of the specimens was performed both in the field and by using “Flora of Ethiopia and Eritrea.” Plant specimen collection and preparation were made using the methods found in [19].

### 2.5. Data Analysis

#### 2.5.1. Informant Consensus

Informant consensus factor (ICF) was computed to measure the level of homogeneity of the information collected and overall agreement on the treatment of specific health disorder category and to identify potential medicinal plant species used for the traditional treatment of human disease categories in the two districts by using the method found in [18]. During information gathering, the informants were contacted three times for the same ideas to determine the reliability of information recorded during the first interview, and the information that was repeated in the same manner by the informants at three contact times was recorded as stated in [19]. ICF was computed as follows: ICF = Nur–Nt/(Nur–1), where Nur is the number of use reports from informants for a particular plant use category, Nt is the number of species that is used for that plant use category for all informants. ICF values range between 0 and 1, where “1” shows the highest level of informant consent [18].

#### 2.5.2. Fidelity Level

The relative healing potential of each reported medicinal plant used against diseases was also tested using an index of fidelity level (FL), and it was calculated as follows: FL (%) = (Ip/Iu) × 100. Here, Ip is the number of informants who independently cited the importance of a species for treating a particular disease, and Iu is the total number of informants who reported the plant for any disease [19].

#### 2.5.3. Preference Ranking

A preference ranking exercise was conducted to rank the medicinal plant that was the most preferred for a particular disease type. In this exercise, the medicinal plant which participants thought to be most effective in treating the reported diseases got the highest value, whereas the one with the least effectiveness got the lowest value (1) [17]. Based on the total score of each species, the rank was determined. This assisted in determining the most effective plant used by the community to treat the most commonly reported diseases.

#### 2.5.4. Direct Matrix Ranking

A direct matrix ranking exercise was also used to test the use diversity of multipurpose medicinal plants of the sampled study areas, as stated in [20]. The exercise also aided in identifying which of the multipurpose plants is most under pressure in the area and to identify the factors which are the threats of medicinal plants. The participants in this exercise were selected based on their long years of experience as traditional herbal medicine practitioners [19] in the districts. For the exercise of direct matrix ranking, a focus group discussion (FGD) was conducted to know the preference based on multipurpose criteria on the plants.

#### 2.5.5. Use Diversity

The collected data were all documented to assess overall use values and diversity of species following [18]. All informants of the study were interviewed at the same time for the use diversity of medicinal plants following the method found in [17, 20]. The local importance of some representative medicinal plants was calculated by using the use value technique (UV). The use value was calculated using the formula UV = ∑Ui/*n* [18], where Ui is the number of uses mentioned by each informant for a species, and *n* is the total number of informants.

#### 2.5.6. Significant Test

The significance of traditional knowledge difference (on the medicinal plants and the disease treated) between general and key informants, adults (≤50 years) and elders (>50 years), female and male, and illiterate (including church and elementary education) and literate was compared using a statistical test and one way ANOVA at 95% confidence level by using SPSS version 20 following the method used in [19].

## 3. Results

### 3.1. Traditional Medicinal Plants

More than one hundred species of medicinal plants were reported to have been used for the traditional treatment of human diseases. The species were recorded under 101 genera and 62 families of angiosperms. Out of the reported species, 8 species were endemic, while 19 species were exotic. Fabaceae was represented by a high species number (8 species, 7.5%), followed by Lamiaceae and Cucurbitaceae (6 species, 5.4% each) and Asteraceae, Euphorbiaceae, and Solanaceae (5 species, 4.5% each). Polygonaceae and Rosaceae (4 species, 3.6% each) and Rubiaceae and Rutaceae (3 species, 2.7% each) were recorded with over 3 species representation while 11 families, namely, Acanthaceae, Alliaceae, Amaranthaceae, Apocynaceae, Brassicaceae, Loranthaceae, Malvaceae, Myrtaceae, Oleaceae, Poaceae, and Ranunculaceae were recorded with 2 species (1.8% each) representation. Each of the remaining 41 families (66.1% of the total families) had a single species (Table 1).

**Figure 2:**
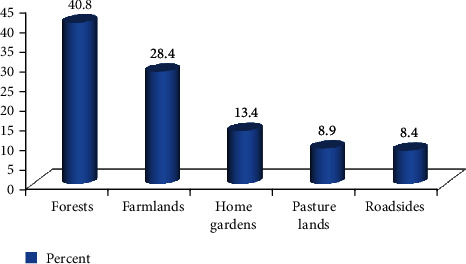
Proportion for collection sites of medicinal plants.

Traditional medicinal plants were recorded with several growth forms. Most of the species were herbs (51 species, 45.5%), followed by shrubs (40 species, 35.7% each), trees (13 species, 11.6%), climbers (including woody and herbaceous climbers) (6 species, 5.4%), and parasitic epiphytes (2 species, 1.8%). The major sites of the collection were bare lands, farmlands, forests, home gardens, irrigation lands, pasture lands, and roadsides. Most of them (73 species, 40.8%) were reported to be collected from forests, while farmlands were the second reported sites of collection of more species (40 species, 22.3%) (Figure 2).

### 3.2. Diagnosis and Treatment Methods

Sixty-one disease types that affect humans were identified to have been treated by traditional medicinal plants. Blood pressure and asthma were found to be the most commonly reported human health problems. Visual inspection and interviews were the most commonly reported diagnosis methods for humans prior to any herbal medicine prescription. Depending on the diseases reported, traditional healers interview patients for symptoms, followed by a visual inspection of eyes, skin color, tongue, throat, the status of sores, bleeding, and infections, and sensing body temperature of their patients with their bare hands. Patients with swellings on the body were reported to have been treated by rubbing and pasting herbal preparations, whereas those with visible wounds were treated by dropping the squeezed liquid on the wound or by tying with a piece of cloth or the patients were ordered to chew the medicinal plant parts for tonsillitis or throat wound or abdominal illness.

### 3.3. Parts Used

The bark, bud, bulb, fruit, latex, leaf, resin, rhizome, root, seed, stem, and flower of traditional medicinal plants were reported to be sources of traditional remedy (medicine). Despite mentioning many plant parts being used for remedy preparation, most of the preparations were from leaves followed by roots and fruits. This possesses about 80% of the remedy. The remaining 20% of the remedy was prepared from the stem (4.1%), bud (4.1%), bark (3.4%), latex (2.8%), flower (2.1%), bulb (1.4%), rhizome (1.4%), and resin (0.7%). Most of the remedies (87.6%) were prepared from freshly harvested parts, whereas about 12% of the remedies were prepared from dried parts.

### 3.4. Preparation and Application

The traditional remedies were reportedly prepared through various ways based on the disease to be treated, the medicinal plants used, and the parts of the plants collected. Thus, most of the remedies used pounding, including splicing (30.2%), and followed by crushing (29.3%) and boiling (distillation) (20.7%). The remaining remedies were prepared by squeezing (8.6%), burning, including fumigating and smoking (5.2%), cooking (3.4%), heating (0.9%), melting (0.9%), and roasting (0.9%). In connection with this, most of the remedies from dried parts were prepared by pounding, while the remedies from freshly harvested parts were prepared by crushing. After a remedy was prepared by such methods, it was reportedly applied to the patients by painting (18.7%), dropping (4.7%), tying (1.9%), washing (0.9%), rubbing (0.9%), or inserting (2.8%). Most (44.9%) of the remedies were applied orally (by drinking), whereas inhaling, chewing, eating, and rinsing were the other application methods of the remedies.

### 3.5. Routes of Administration, Dosages, and Antidotes

A prepared traditional remedy (medicine) is reportedly administered through five routes. However, most of the remedies (59.6%) were administered through oral routes followed by dermal routes (24.8%) and nasal routes (10.1%). The remaining remedies were administered through auricular and optical routes (2.8% each) depending on the disease. A remedy was reportedly given to patients by measuring it using traditional standards of measurement such as the amount in a cup (at the depth of a joint of a finger), a spoonful, and a glass of tea (if it is in liquid form). However, as it was reported, the amount of a remedy is also determined by age, pregnancy status, and physical appearance of the patient. Sulfur, eye cosmetics, salt, honey, malt, water, butter, milk, buttermilk, stew, myrrh, and Katicala and local liquor were the commonly reported antidotes for herbal preparations with adverse side effects.

### 3.6. Marketability

Twenty-three medicinal plants were reportedly sold in local marketplaces of Adet and Dabi and in other small markets of the districts. These species were grouped under 18 families. Asteraceae has three species (*Helianthus annuus*, *Guizotia abyssinica*, and *Echinops kebericho*), Fabaceae has two species (*Lupinus albus* and *Trigonella foenum-graecum*), Alliaceae has two species (*Allium cepa* and *A. sativum*), and Rutaceae has two species (*Citrus aurantiifolia* and *Ruta chalepensis*). The other 14 families were represented by a single species each. The species were *Amaranthus caudatus*, *Brassica carinata*, *Carica papaya*, *Catha edulis*, *Cofea arabica*, *Hordeum vulgare*, *Laggera siceraria*, *Linum usitatissimum*, *Myrtus communis*, *Nigella sativa*, *Olea europaea* subsp. *cuspidata*, *Otostegia integrifolia*, *Securidaca longepedunculata*, and *Zingiber officinale*. *Echinops kebericho*, *Myrtus communis*, *Olea europaea* subsp. *cuspidata*, *Otostegia integrifolia*, and *Securidaca longepedunculata* were sold entirely for medicinal applications. Approximately 7 cm of the root of *Echinops kebericho* costs six Birr (Ethiopian currency), and a handful of leaves of *Myrtus communis* costs 3 Birr. Similarly, a bunch of *Olea europaea* subsp. *cuspidata* and *Otostegia integrifolia* was three Birr. A small piece of a *Securidaca longepedunculata* dried stem was six Birr. The remaining reported medicinal plants were mainly sold in bulk for their nonmedicinal uses, though they are rarely applied as a source of traditional medicine when they are needed.

### 3.7. Informants' Agreements

The central nervous system and digestive system diseases were recorded with the highest informant consensus values (0.9 each), followed by dermal and infectious diseases (0.8). More species of medicinal plants were cited for the latter two disease categories (Table 2).

### 3.8. Relative Healing Potential

*Dodonea angustifolia*, *Dovyalis abyssinica*, *Hagenia abyssinica*, *Nigella sativa*, and *Urtica simensis* were recorded with the highest healing potential value for malaria, asthma, tapeworm, cough, and stomach ulcer, respectively (FL = 100) (Table 3).

### 3.9. Use Preference

*Verbascum sinaiticum* was recorded with the highest total score (TS = 74) for traditional treatment of blood pressure, while *Thymus schimperi* was the second with a total score of 70 for blood pressure (Table 4). As shown in Table 5, *Verbascum sinaiticum* was further recorded as the most preferred species for treating asthma (TS = 45), followed by *Catha edulis* (TS = 44), whereas *Olea europaea* subsp. *cuspidata* was ranked first (most threatened), followed by *Carissa spinarum* (Table 6).

### 3.10. Use Values

*Trigonella foenum-graecum* was recorded with the highest use value (UV = 6.8), followed by *Carissa spinarum* (UV = 4.7) (Table 7). Fifteen medicinal plants (8.5%) were reported to have been used only for traditional medicine, whereas most medicinal plants (97 species, 86.5%) were cited for one or more uses other than their medicinal role. *Cucumis ficifolius* was a highly cited species (cited by 135 informants), followed by *Zehneria scabra* (cited by 22 informants). Regarding their use values as traditional medicine, better medicinal use values (UVmed) were recorded for *Rumex nepalensis* (3.5) and *Lepidium sativum* (1) (Table 8).

### 3.11. Use Diversity

**Figure 3:**
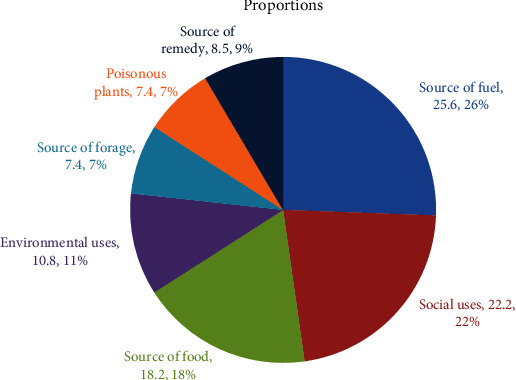
Proportions of medicinal plants under various use categories.

The medicinal plants can be assigned into various use categories (Figure 3).

#### 3.11.1. As Source of Food

Thirty-two (18.2%) medicinal plants were reportedly used as a source of food (including spices) for humans. These species were grouped under 28 genera and 25 families. Lamiaceae had good species representation of (represented by 3 species, 9.4%), followed by Rosaceae, Polygonaceae, Asteraceae, Apiaceae, and Alliaceae (2 species, 6.3% each). The remaining families were represented by a few medicinal plant species (1 species, 3.1% each).

#### 3.11.2. Forage Plants

Thirteen species (7.4%) of medicinal plants were reportedly used as fodder for livestock and bees. These species were grouped under 13 genera and 9 families. Asteraceae had 3 representing species (23%), followed by Fabaceae and Poaceae with 2 representing species (15.4% each). The remaining 10 families were represented by a single species (7.7% each). The species under this category were recorded with 3 habit types, namely trees (4 species, 30.8%), shrubs (1 species, 7.7%), and herbs (8 species, 61.5%).

#### 3.11.3. Fuel

Forty-five (25.6%) species of medicinal plants were reportedly used for firewood. The seeds of *Ricinus communis* were reportedly used to prepare a traditional kerosene lamp. The species were grouped under 44 genera and 27 families. Fabaceae had the highest species representation (6 species, 13.3%), followed by Asteraceae, Euphorbiaceae, and Lamiaceae (3 species, 6.7% each). Individually, Apocynaceae, Myrtaceae, Oleaceae, Polygonaceae, Rutaceae, and Rosaceae had 2 representative species (4.4%). The remaining 17 families had a single representative species (2.2% each). Most of the species (82.2%) were woody species, whereas the remaining 17.8% of the total species were herbs.

#### 3.11.4. Poisonous

Thirteen species (7.4%) are poisonous if they come in contact with the body. These species were toxic for humans, livestock, and other animals. They have potential as insecticides and insect repellents. *Acokanthera schimperi*, *Argemone mexicana*, *Euphorbia abyssinica*, and *Euphorbia platyphyllos* were reported to have toxic latex for humans. *Solanum anguivi*, *S. incanum*, *S. marginatum*, *Arisaema schimperianum*, and *Cyphostemma cyphopetalum* were reported to have toxic fruits. The other reported toxic parts of medicinal plants were the leaves of *Eucalyptus globulus* and the seed of *Ricinus communis*. These parts were reportedly toxic if they were taken in through the mouth of humans.

#### 3.11.5. Social Uses

**Figure 4:**
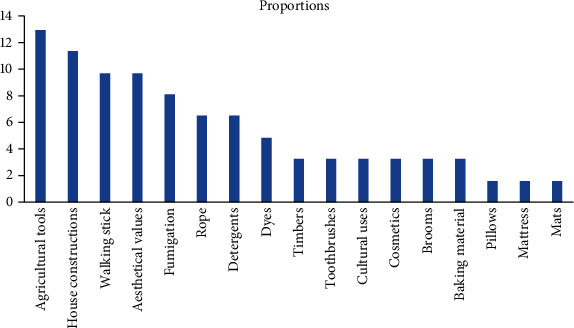
Proportions (%) of medicinal plants under various social uses.

Thirty-nine (22.2%) species were reportedly used as a source of stimulants, fumigation, cosmetics, walking stick, baking materials, agricultural tools, house construction materials, dyes, pillow, mattress, toothbrush, rope, ornamental, mat, cultural uses, detergents, and timber (preparation of beds, tables, and other household utensils). These species were grouped under 37 genera and 24 families. Euphorbiaceae and Fabaceae had the highest species representation (4 species, 10.3% each), followed by Asteraceae and Lamiaceae (3 species, 7.7% each). Malvaceae, Myrtaceae, Poaceae, Rosaceae, and Rutaceae were represented by 2 species (5.1% each). The remaining 30 families were individually represented by a single species (2.6% each). Most of the total species (13%) were reportedly used for agricultural tools followed by those that were used for house construction (Figure 4).

#### 3.11.6. Environmental Uses

Thirty-three (10.8%) of the medicinal plant species were reportedly used for environmental purposes (live fence, dry fence, shades, and erosion control and soil improvement). These were distributed in 31 genera and 22 families. Fabaceae had the highest species representation (5 species, 15.2%), followed by Asteraceae and Solanaceae (3 species, 9% each). Acanthaceae, Euphorbiaceae, and Poaceae were ranked third in species representation (2 species, 6% each). The remaining 16 families were represented by a single species (1 species, 3% each). Most of the species were recorded from herbs and shrubs (15 species, 44.1% each). However, trees were recorded with the least number of species (4 species, 11.8%). Out of the total species of the group, 20 species (64.7%) were reportedly used to control erosion and to improve soil fertility. The remaining 14 (35.3%) species were reportedly used for fencing (live fence, dry fence) and shades.

### 3.12. Traditional Knowledge Difference and Transfer

#### 3.12.1. Traditional Knowledge Difference

One way ANOVA and *F* test on the variance of medicinal plant knowledge difference between and among informant categories were performed. Following the analysis, there was a significant knowledge difference (*p* < 0.05) between general and key informants, adults and elders, illiterates and literates, and between males and females (Table 9).

#### 3.12.2. Traditional Knowledge Transfer

Traditional knowledge was reported to be transferred from church teachers to church students and from parents to children, but rarely was it reportedly gained from friends. It was reported that most informants (359 informants, 90.9%) gained their knowledge from their parents, whereas the TK of some informants was reportedly gained from church teachers.

#### 3.12.3. Threats of Traditional Knowledge

The major reported threats for losing traditional knowledge of the local people include a decrease in the number of church students; the expansion of modern pharmacies, which led the customers of the healers to ignore traditional medicine; the expansion of modern education, which led youngsters to ignore the traditional schools (churches, reportedly the major reservoirs of traditional knowledge); scarcity or low availability of traditional medicinal plants in the surrounding areas; and the migration of traditional healers to towns or cities (Table 10).

#### 3.12.4. Threats and Conservation Status

The major reported threats of medicinal plants in the current study areas are agricultural expansion, including clearance of the major habitats of the species, and low attention of the local communities in conserving and protecting the species. However, there were reportedly indirect conservation activities of the species. These included the planting of the species for indirect uses such as aesthetics, fence, food, shade, spice, fuelwood, and source of income. Seasonal protection of major habitats of the species was reported as a recent conservation activity by the local people in these areas.

## 4. Discussions

### 4.1. Traditional Medicinal Plants

The result showed that all medicinal plant species belong to angiosperms. This also agreed to the results in [21] as almost all traditional medicinal plants were described as flowering plants. This might be because of the success of flowering plants in invading large areas of the globe, and it might also be because of their huge diversity of species. Ethiopia is one of the six countries in the world wherein about 60% of the plants found therein are said to be indigenous with healing potential [22]. Out of the c. 213 families of flowering medicinal plants in Ethiopia, 92 families have medicinal properties [23].

The results showed that Fabaceae has several medicinal plant records (8 species) compared to the other families in the current study areas. In agreement with the current study, other findings also recorded Fabaceae as the dominant family in these areas in terms of species number [24]. However, other research findings disagree with the result of the current study areas, and they recorded Asteraceae as the dominant family of their study areas [25]. The dominance of Fabaceae as a medicinal plant might be because of its content of active flavonoids that make it unique from other families. This is because isoflavonoids are common in species of Fabaceae but are found in few other plant families [26].

The results also showed that in most species, medicinal plants come from herbs followed by shrubs. In agreement with the current study areas, other research findings also reported herbs as the primary source of remedy in other parts of Ethiopia [27]. The findings that herbaceous species are the primary source of traditional medicines in the current study districts might be related to the fact (1) that they are more easily accessible in the nearby areas than are trees and shrubs; (2) they are more abundant because of a relatively high amount of rainfall in the areas; and (3) they are rich with strong bioactive compounds, meaning herbs contain phytochemicals like alkaloids and flavonoids that have strong antibacterial and antifungal effects [28].

### 4.2. Comparison of Species Record to Some Other Parts of Ethiopia

The results showed that the current study areas are rich in medicinal plants compared to the other parts of Ethiopia such as the Babile district (51 species) [6], the Amaro district (56 species) [29], the Enderta district (27 species), and the Alamata district (25 species) [30]. It further shows that the current study districts have comparable medicinal plant records compared to some other parts of the country such as the Ankober district (135 species) [24], the degraded land of Tigray (259 species) [31], the Wayu Tuka district (126 species) [32], and the Kilte Awulaelo district (114 species) [33]. The difference in the number of medicinal plant records might be because of the study area coverage, the difference in the number of key informants, and the difference in plant diversity and culture of the study areas.

### 4.3. Types of Diseases/Ailments

The results of the current study show that blood pressure and asthma are the prevalent human health problems in these areas. According to the results in [34], the treatment of asthma using modern medicine have not suppressed all of its symptoms. Thus, it showed that treating this disease by medicinal plants is highly efficacious, and it is low in cost, easy to manage, and has few adverse effects [35]. It also showed that leaves are the primary source of remedy. According to the findings in [36], the extracts from many parts of plants such as the barks, flowers, fruits, leaves, and roots of medicinal plants have secondary compounds such as alkaloids, naphthoquinones, saponins, flavonoids, terpenoids, sesquiterpenoids, quassinoids, xanthones, and ferruginol that have ethnopharmacological uses. Thus, different parts of medicinal plants have curative properties for ailments. However, the use of leaves as primary sources of remedy might be related to conservation and sustainable utilization, ease of preparation and collection, and availability of medicinal plants in nearby surrounding areas. In agreement with the current study, similar results of using leaves as a primary source of remedies were reported in other parts of Ethiopia [37]. The results showed that the availability and marketability of medicinal plants are quite low in local markets of the districts. It further showed that even the available medicinal plants are mostly sold in bulk for their nonmedicinal purposes. However, a few numbers of species are sold for their medicinal role. This low availability and marketability of medicinal plants in local markets might indicate a low culture of selling and buying medicinal plants. It further showed that most of the medicinal plants used for remedy preparation are fresh, not dried. This is because most of the medicinal plants found in the local markets are dry. In addition, it showed that the medicinal plant trade, which is one cause why medicinal plants are endangered in other parts of the world, is not a threat for medicinal plants in these areas [38].

### 4.4. Agreements of Informants

The results showed that miscellaneous, central nervous system and digestive system diseases have the highest informant consensus values (0.9 each). It also showed that dermal and infectious diseases have the second-highest informant agreements (0.8 each). According to the findings in [28], ICF values are always greater when a single plant or a few plants are documented to have been used by many of the respondents to cure a specific disease, while low ICF values show that informants do not agree over which plant to use. The highest informant agreements show the prevalence of the disease in the areas. Thus, the result showed that the diseases with the highest ICF were prevalent in the areas. Especially, the highest occurrence of digestive system diseases in the districts might be because of poor hygiene, fuelwood smoke inside houses, water, and air pollution. It further showed that high ICF values indicate that these diseases are more prevalent in these districts because of the poor socioeconomic and sanitary conditions of the people [39].

### 4.5. Healing Potential and Use Value

The results prove that out of a total of 14 medicinal plants, *Dodonea angustifolia*, *Dovyalis abyssinica*, *Hagenia abyssinica*, *Nigella sativa*, and *Urtica simensis* have the highest fidelity level. According to the findings in [40], medicinal plants with the highest fidelity level indicate that they are widely used by the local people while those with lower fidelity levels are not widely used. Thus, it shows that these species are the most widely used species in the current study areas. The highest fidelity level shows the distribution of the knowledge of the species among the local people [18]. The results show that out of a total of 24 of the most cited traditional medicinal plants, *Trigonella foenum-graecum* has the highest use value (UV = 6.8), followed by *Carissa spinarum* (UV = 4.7). According to the findings in [41], the relative importance of plants reflects the number of uses assigned to it. The results using this technique are commonly interpreted as the pressure on a resource resulting from use, considering the logic that the most well-known resources are also the most used. However, there are no studies that have established a direct relationship between use value and the real pressure on a resource resulting from the use. A plant with a high use value would theoretically have a correspondingly high cultural consensus [18].

### 4.6. Use Preference

The findings of the current study from the preference ranking of 7 medicinal plants used to treat blood pressure showed that *Verbascum sinaiticum* is the most preferred medicinal plant with a total score of 74. It further shows that this mentioned species is the most preferred for asthma with a total score of 45. The other preference ranking exercise for 6 selected medicinal plants used to treat common cold shows that *Otostegia integrifolia* is the most preferred one with a total score of 64. According to the findings in [42], such types of species with the highest scores show that the species are under the highest extract pressure even though there are several agents acting in the selection of a specific plant that can be related to cultural and ecological issues and the intrinsic characteristics of a species.

### 4.7. Use Diversities

The results showed that out of the total recorded medicinal plants in these areas, the majority (97 species, 86.5%) were cited for one or more uses other than their medicinal role showing that most of the species are exposed to the highest threats. It also showed that medicinal plants such as *Olea europaea* subsp. *cuspidata*, *Carissa spinarum*, and *Rosa abyssinica* with the highest total score of 95, 84, and 76, respectively, are the most widely used species by the local community in these areas. Based on the direct matrix ranking concept, such species are the first threatened species in the areas because of their diverse use. The results further showed that *Olea europaea* subsp*. cuspidata* has several uses for agricultural tools well over other woody species because of the strength of its wood. It also showed that this species is not widely distributed. So, many of the local communities depended on this species for this purpose. In addition, it is a preferred plant for fumigation, so its branches are widely sold in the local markets of the districts. Thus, the species is the most threatened species.

Like in other areas of Ethiopia, agricultural expansion is the major threat of the multipurpose species in the Yilmana Densa and Quarit districts [43, 44]. However, in contrast to other findings, overharvesting is also reported as the other major threat for the multipurpose species of the current districts. This might be because the method of their utilization or harvesting for such purposes might kill the roots (*Carissa spinarum* roots are the source of medicine) and might be a severe threat to the survival of rare and slowly reproducing medicinal plants of the areas [24]. This might be also one reason for the local community to primarily use the leaves as their primary source of traditional herbal medicine. Firewood is also the other major threat of woody species in other parts of Ethiopia in agreement with the findings of the current study [45, 46].

### 4.8. Traditional Knowledge Difference and Transfer

The results further showed that the local communities of the study areas are endowed with traditional knowledge with a significant difference (*p* < 0.05) between general and key informants, adults and elders, illiterates and literates, and males and females. In agreement with the current study, such types of significant knowledge differences between informant groups were reported by other research findings in other parts of Ethiopia. The presence of significant traditional knowledge differences between general and key informants, adults, and elders was recorded in [47], that between illiterates and literates was recorded in [25], and that between males and females was recorded in [25, 48].

According to the findings in [6], the traditional knowledge difference is due to the fragmentation and erosion of the traditional knowledge from illiterate healers because of the absence of formal written records used as a reference. Key informants might also upgrade and develop their knowledge from their daily trial-and-error experience or from the experience of other practitioners, since it might be their livelihood. Similarly, elders might have good experience because of their age. Besides, they might have experience in the use of many medicinal plants that have existed in previous years, but have nowadays vanished from the surrounding areas. Age and experience might also be the reason for the significant knowledge difference between elders and adults as recorded in other parts of Ethiopia [24]. Males have better TK than females. This might be because males probably have experience with multiple sources of knowledge. Males have the experience of church education, while females might not; males might usually perform their day-to-day activities outside their houses, but females might usually do their activities inside the house. So this might help males acquire TK from their friends, since they might meet more friends than females outside their houses.

However, the results show that the traditional knowledge gained (9.1%) from these sources is much lower than that gained from parents (90.9%). The percentage difference of TK gained from different sources might be because of doubt between TK donors and receivers. According to the findings in [44], the secrecy of traditional medical practice is a common phenomenon elsewhere in Ethiopia. Thus, children might be more loyal to parents than are other groups, so parents might transfer their knowledge in a more comprehensive manner to their children than they would to others. Knowledge might be transferred from parents to children without payment, but with significant payment to those of outside the family as stated in other research works [49].

## 5. Conclusions

The study districts are the rich sources of medicinal plants as they are the major sources of medicines in the districts. Thus, the local people of the districts have reserved indigenous knowledge on medicinal plants. Nowadays, minimal attention has been given to the use and conservation of medicinal plants. Most of the species are nonmanageable and nonmarketable. Therefore, the creation of extensive awareness is needed regarding the management and conservation of medicinal plants and the habitats which are the home for most of the species. It also important that there is coordination established between the traditional healers and the district's health offices for the successful provision of traditional medicines.

## Figures and Tables

**Table 1:** List of medicinal plants used for the traditional treatment of human diseases/ailments: scientific name, family, local name, parts used, disease treated, mode of preparation, and routes of administration.

Scientific name	Amharic name	Parts used	Disease treated	Mode of preparation	Administration
*Acacia negrii* Pic. Sermi. (Fabaceae)	Cheba	Resin	Wound	Painting	Dermal
*Achyranthes aspera* L. (Amaranthaceae)	Telenji	Leaf	Throat (esophagus) disease	Cutting and crushing	Oral
*Acokanthera schimperi* (DC.) Oliv. (Apocynaceae)	Merenz	Leaf, stem	Syphilis, wound	Pounding and painting	Dermal
*Allium cepa* L. (Alliaceae)	Qey Shnkurt	Bulb	Headache	Eating	
*Allium sativum* L. (Alliaceae)	Nech Shnkurt	Bulb	Evil eye, cough, abdominal pain (vomiting), swelling, malaria	Crushing and eating	Oral
*Amaranthus caudatus* L. (Amaranthaceae)	Yebahir Teff	Root, stem	Frequent miscarriage/neonatal death	Pounding	Oral
*Argemone mexicana* L. (Papaveraceae)	Dendero	Fruit	Wound	Crushing and dropping	Dermal
*Asparagus africanus* Lam. (Asparagaceae)	Yeset Kest	Root	Migraine	Binding	Dermal
*Astragalus atropilosulus* (Hochst.) Bunge (Fabaceae)	Yebab Alenga	Root	Snake bite	Chewing	Oral
*Brassica carinata* A. Br. (Brassicaceae)	Gomenzer	Seed	Abdominal pain	Pounding	Oral
*Brucea antidysenterica* J. F. Mill. (Simaroubaceae)	Abalo/W-aginos	Leaf, fruit	Madness/mental disease	Eating	Oral
*Buddleja polystachya* Fresen. (Loganiaceae)	Anfar	Leaf, root	Headache	Pounding	Nasal
*Carica papaya* L. (Caricaceae)	Papaya	Leaf	Diabetes	Boiling	Oral
*Carissa spinarum* L. (Apocynaceae)	Agam	Leaf, root	Snake bite, mental illness, toothache	Chewing or crushing	Oral
*Calpurnia aurea* (Ait.) Benth. (Fabaceae)	Ligita	Root, leaf, bark	Snake bite	Chewing	Oral
*Catha edulis* (Vahl) Forssk. ex Endl. (Celastraceae)	Chat	Leaf	Malaria, cough, asthma	Boiling	Oral
*Citrus aurantiifolia* (Christm.) Swingle. (Rutaceae)	Lomi	Fruit	Gonorrhea	Crushing	Oral
*Clausena anisata* (Willd.) Benth. (Rutaceae)	Limich	Leaf, fruit	Madness/mental illness	Chewing	Oral
*Clerodendrum myricoides* (Hochst.) Vatke (Lamiaceae)	Misirch	Leaf	Migraine	Crushing	Oral
*Clutia lanceolata* Jaub. & Spach. (Euphorbiaceae)	Fiyele feji	Root	Frequent miscarriage/neonatal death	Pounding	Oral
*Coccinia abyssinica* (Lam.) Cogn (Cucurbitaceae)	Werq Bemieda	Root	Malaria, evil eye	Crushing	Oral
*Coffea arabica* L. (Rubiaceae)	Buna	Stem	Headache	Roasting	Nasal
*Crotalaria spinosa* Hochst. ex Benth. (Fabaceae)	Yemdir Grar	Root	Rabies	Boiling	
*Croton macrostachyus* Del. (Euphorbiaceae)	Bisana	Latex, leaf, bud	Tinea nigra, Tinea corporis, blood pressure, snake bite, eye disease, epistaxis (nose bleeding), malaria	Crushing, squeezing	Nasal, optical
*Cucumis ficifolius* A. Rich. (Cucurbitaceae)	Yemdir Embuay	Root, leaf	Evil eye, abdominal pain (bloating, vomiting), febrile illness, rabies, ear pest	Crushing and cooking	Auricular
*Cucurbita pepo* L. (Cucurbitaceae)	Duba	Seed	Rheumatic pain	Pounding	Oral
*Cynoglossum coeruleum* Hochst. ex A. DC. (Boraginaceae)	Shemigeg/Etse-demena	Leaf, root	Febrile illness, spider bite, toothache	Crushing, painting, boiling and chewing	Nasal, dermal, oral
*Cyperus rigidifolius* Steud. (Cyperaceae)	Angicha	Root/stolon	Snake bite	Pounding	Oral
*Cyphostemma cyphopetalum* (Fresen.) Descoings ex Wild & Drummond (Vitaceae)	Yejib Areg	Fruit	Poisonous	Chewing	Oral
*Datura stramonium* L. (Solanaceae)	Astenagir	Leaf, seed	Dandruff, rheumatic pain	Crushing and smoking	Nasal
*Dodonea angustifolia* L.f. (Sapindaceae)	Kitikita	Root, leaf	Malaria	Crushing	Oral
*Dovyalis abyssinica* (A. Rich.) Warb. (Flacourtiaceae)	Koshim	Leaf	Blood pressure, asthma	Boiling	Oral
*Echinops kebericho* Mesfin (Asteraceae)	Kebericho	Root	Evil spirit, evil eye, mental illness, febrile illness, common cold	Burning, smoking pounding	Nasal
*Embelia schimperi* Vatke (Myrsinaceae)	Enkoko	Fruit	Ascaris, abdominal bloating, Taenia saginata infection or beef tapeworm	Pounding	Oral
*Eucalyptus globulus* Labill. (Myrtaceae)	Bule Bahir Zaf	Leaf	Common cold	Boiling	Nasal
*Euclea racemosa* Murr. subsp. *schimperi* (A. DC.) White (Ebenaceae)	Dedeho	Leaf	Eczema	Pounding	Dermal
*Euphorbia abyssinica* Gmel. (Euphorbiaceae)	Kulkual	Latex	Rabies	Cutting	Dermal
*Euphorbia platyphyllos* L. (Euphorbiaceae)	Abaydem	Latex	Poisonous	Painting	
*Ferula communis* L. (Apiaceae)	Inslal	Leaf	Night blindness	Crushing and painting	Optical
*Ficus palmata* Forssk. (Moraceae)	Qotilebele-s	Latex, leaf	Breast swelling, swelling	Boiling and dropping	Dermal
*Gladiolus abyssinicus* (Brongn. ex Lemaire) Goldblatt & de Vos (Iridaceae)	Enzeresey	Root	Hemorrhoids	Pounding then add to the hemorrhoids	Dermal
*Guizotia abyssinica* (L. f.) Cass. (Asteraceae)	Nug	Seed	Tinea capitis (boldness)	Pounding and painting	Dermal
*Guizotia schimperi* Sch. Bip. ex Walp. (Asteraceae)	Mech	Root, leaf	Ascaris, Taenia saginata infection or beef tapeworm	Pounding and drinking	Oral
*Hagenia abyssinica* (Bruce) J. F. Gmelin (Rosaceae)	Koso	Fruit	Ascaris, Taenia saginata infection or beef tapeworm	Pounding/crushing	Oral
*Helianthus annuus* L. (Asteraceae)	Suf	Seed	Rabies	Pounding	Oral
*Hygrophila schulli* (Hamilt.) M.R. & S.M. (Acanthaceae)	Amekela	All parts	Eczema	Burning	Dermal
*Impatiens ethiopica* Grey-Wilson^∗∗^ (Balsaminaceae)	Insosla	Root	Cough	Crushing and pounding	Oral
*Jasminum abyssinicum* Hochst. ex DC. (Oleaceae)	Tenbelel	Leaf, root	Ascaris, Taenia saginata infection or beef tapeworm	Pounding	Oral
*Justicia schimperiana* (Hochst. ex Nees) T. Anders. (Acanthaceae)	Sensel	Root, leaf	Abdominal pain (bloating), swelling, rabies	Crushing, squeezing	Oral
*Kalanchoe petitiana* A. Rich. (Crassulaceae)	Andahula	Root	Tonsillitis	Crushing	
*Kanahia laniflora* (Forssk.) R. Br. (Asclepiadaceae)	Tifrena	Latex, root	Gland removal, evil eye, scabies (itching)	Crushing, dropping, painting	Dermal
*Laggera crispata* (Vahl) Hepper & Wood (Asteraceae)	Kes Bedeje	Root	Abdominal burning (gastritis) and knocking	Cooking, eating	Dermal
*Laggera siceraria* (Molina) Standley (Cucurbitaceae)	Qil	Fruit	Abdominal bloating, stomachache	Pouring and shaking	Oral
*Leonotis ocymifolia* (Burml. f.) Iwarsson var. *raineriana* (Visiani) Iwarsson (Lamiaceae)	Ras Kimr	Root, flower	Cancer, wound	Crushing	Dermal
*Lepidium sativum* L. (Brassicaceae)	Feto	Seed	Febrile illness, evil eye, gonorrhea	Pounding, drinking	Oral
*Linum usitatissimum* L. (Linaceae)	Telba	Seed	Constipation, eye disease	Pounding, inserting	Oral, optical
*Lobelia rhynchopetalum* Hemsl. (Lobeliaceae)	Gibra	Root	Impotency	Crushing	Oral
*Lupinus albus* L. (Fabaceae)	Gibto	Seed	Blood pressure	Pounding, cooking	Nasal
*Melia azedarach* L. (Meliaceae)	Nim	Leaf	Malaria	Crushing, drinking	Oral
*Millettia ferruginea* (Hochst.) Bak. (Fabaceae)	Birbira	Seed	Tinea nigra, Tinea corporis, eczema, skin peeling	Pounding, painting	Dermal
*Momordica foetida* Schumach. (Cucurbitaceae)	Kurgn	Leaf	Wound	Squeezing	Dermal
*Myrica salicifolia* A. Rich. (Myricaceae)	Shinet	Bark	Migraine	Pounding	Nasal
*Myrtus communis* L. (Myrtaceae)	Ades	Leaf	Wound, scabies (itching)	Crushing and painting	Dermal
*Nigella sativa* L. (Ranunculaceae)	Tikur Az-mud	Bulb, seed	Cough	Boiling and drinking	Oral
*Ocimum americanum* L. (Lamiaceae)	Ziqaqibie	Leaf, flower	Lung disease, liver disease/jaundice, intestinal diseases	Drinking	Oral
*Ocimum urticifolium* Roth (Lamiaceae)	Dama Ke-sie	Leaf, fruit	Febrile illness, abdominal pain, common cold	Crushing, painting, boiling	Oral, dermal
*Olea europaea* L. subsp. *cuspidata* (Wall. ex G. Don) Cif. (Oleaceae)	Woira	Leaf, stem	Common cold, night blindness	Burning, squeezing	Nasal, optical
*Olinia rochetiana* A. Juss. (Oliniaceae)	Tifie	Stem	Swelling	Chewing	Dermal
*Osyris quadripartita* Decn. (Santalaceae)	Keret	Root	Trachoma	Crushing	Optical
*Otostegia integrifolia* Benth. (Lamiaceae)	Tinjut	Leaf	Common cold, evil spirit	Burning	Nasal
*Pennisetum thunbergii* Kunth (Poaceae)	Sindedo	Root, leaf	Febrile illness	Burning	Nasal
*Persea americana* Mill. (Lauraceae)	Avocado	Leaf	Diabetes	Boiling and drinking	Oral
*Phoenix reclinata* Jacq. (Arecaceae)	Selen	Fruit	Deafness	Pounding and boiling	Auricular
*Phragmanthera regularis* (Sprague) M. Gilbert (Loranthaceae)	Teketila	Leaf	Blood pressure	Pounding or crushing, drinking	Oral
*Phragmanthera macrosolen* (A. Rich.) M. Gilbert (Loranthaceae)	Teketila	Leaf	Blood pressure	Pounding or crushing, and drinking	Oral
*Phytolacca dodecandra* L'Her. (Phytolaccaceae)	Endod	Leaf, fruit, root	Abdominal pain (swelling, vomiting), rabies, scabies (itching)	Crushing, pounding, cooking, washing	Dermal, oral
*Piper nigrum* L. (Piperaceae)	Qundo Berbere	Fruit	Hemorrhoids	Pounding	Dermal
*Plantago lanceolata* L. (Plantaginaceae)	Gorteb	Leaf	Wound	Crushing	Dermal
*Plumbago zeylanica* L. (Plumbaginaceae)	Amira	Leaf	Abdominal pain, swelling	Boiling	Oral
*Pterolobium slocal liquortum* (Forssk.) Brenan (Fabaceae)	Keltefa	Root	Evil eye	Crushing or pounding	Nasal
*Punica granatum* L. (Punicaceae)	Roman	Bark	Abdominal pain	Cooking	Oral
*Ricinus communis* L. (Euphorbiaceae)	Chakima	Root, seed	Abdominal impelling, poisoning, abdominal pain	Chewing or crushing	Oral
*Rubia cordifolia* L. (Rubiaceae)	Enchibir	Root, leaf	Asthma	Boiling	Oral
*Rosa abyssinica* Lindley(Rosaceae)	Kega	Fruit	Blood pressure	Boiling	Oral
*Rosa* x *richardii* Rehd. (Rosaceae)	Tsigereda	Flower	TB (head wound)	Boiling	Nasal
*Rubus apetalus* Poir. (Rosaceae)	Enjori	Stem	Blood pressure	Eating	Oral
*Rumex abyssinicus* Jacq. (Polygonaceae)	Mekmeko	Root	Diabetes	Pounding	Oral
*Rumex nepalensis* Spreng. (Po1ygonaceae)	Yewusha Milas	Root	Abdominal impelling, abdominal pain, abdominal swelling, diarrhea, evil eye, tonsillitis	Crushing	Oral
*Rumex nervosus* Vahl (Polygonaceae)	Ambacho	Stem, leaf, bulb	Warts, common cold, wound	Heating, boiling, squeezing, painting	Dermal, oral, nasal
*Ruta chalepensis* L. (Rutaceae)	Tenadam	Flower, stem	Evil eye, abdominal pain, swelling, vomiting	Pounding	Nasal
*Scadoxus multiflorus* (Martyn) Raf. (Amaryllidaceae)	Chiret	Root	TB (wound), abdominal and penis swelling	Pounding, painting, crushing	Dermal, oral
*Schefflera abyssinica* (Hochst. ex A. Rich.) Harms (Araliaceae)	Getem	Root, leaf	Snake bite	Pounding, squeezing	
*Securidaca longepedunculata* Fresen. (Polygalaceae)	Etse-men-ahe	Bark	Headache	Pounding	Nasal
*Sesamum orientale* L. (Pedaliaceae)	Selit	Root, fruit	Hemorrhoids	Pounding	Dermal
*Sida rhombifolia* L. (Malvaceae)	Gorjejit	Leaf	Vomiting, wound	Crushing and painting	Dermal
*Sida schimperiana* Hochst. ex A. Rich. (Malvaceae)	Chifrig	Stem, leaf	Gonorrhea, urinary retention	Boiling	Oral
*Solanum anguivi* Lam. (Solanaceae)	Zerech Embuay	Fruit	Wound	Crushing	Dermal
*Solanum incanum* L.(Solanaceae)	Embuay	Fruit	Scabies (itching), wound	Crushing, painting	Dermal
*Solanum nigrum* L. (Solanaceae)	Awut	Fruit, leaf	Scabies (itching), burned wound	Crushing and painting	Dermal
*Stephania abyssinica* (Dillon & A. Rich.) Walp. (Menispermaceae)	Yedimet Ain	Root	Abdominal impelling	Crushing	Oral
		Leaf	Febrile illness	Boiling	Oral
		Root	Diarrhea	Chewing	Oral
*Thymus schimperi* Ron. (Lamiaceae)	Tosign	Leaf	Blood pressure	Boiling	Oral
*Trigonella foenum-graecum* L. (Fabaceae)	Abish	Seed	Abdominal pain	Pounding and boiling	Oral
			Wound	Pounding and painting	Dermal
			Cough	Cooking and pounding	Oral
*Urtica simensis* Steudel (Urticaceae)	Sama	Leaf	Rheumatism/rheumatism	Boiling	Oral
			Stomach ulcer	Cooking	Oral
*Verbascum sinaiticum* Benth. (Scrophulariaceae)	Daba Keded	Root, leaf	Swelling	Crushing	Oral
		Root	Abdominal pain, vomiting	Chewing	
			Eczema	Crushing	Dermal
			Esophagus swelling	Crushing	Oral
			Asthma	Pounding	Oral
			Nose bleeding (epistaxis)	Pounding	Nasal
			Blood pressure	Chewing	Oral
			Loss of weight	Crushing	Oral
*Verbena officinalis* L. (Verbenaceae)	Atuch	Root, leaf, stem	Poisoning	Chewing	Oral
		Root, leaf, stem	Diarrhea	Crushing	Oral
		Root, leaf	Snake bite	Crushing	Oral
		Leaf	Tongue disease	Boiling	Oral
		Root, leaf, stem	Acute febrile illness (elevated temperature (°C)),	Crushing	Oral
		Root, leaf, stem	Stabbing pain	Crushing	Oral
*Withania somnifera* (L.) Dunal (Solanaceae)	Egiziwa	Leaf	Diarrhea	Crushing	Oral
		Fruit	Hemorrhoids	Crushing	Dermal
*Ximenia americana* L. (Olacaceae)	Enkoy	Root, leaf	Wound	Pounding	Oral
*Zehneria scabra* (Linn. f.) Sond. (Cucurbitaceae)	Areg Resa	Leaf stem	TB (wound)	Squeezing	Nasal
			Febrile illness (fever)	Boiling	Nasal
			Trachoma	Boiling	Nasal
*Zingiber officinale* Roscoe (Zingiberaceae)	Zingibl	Rhizome	Cough, common cold	Crushing and boiling	Oral

**Table 2:** Consensus of informants on different use categories.

Disease categories	Nur	Nt	ICF
(1) Nervous diseases (migraine, headache, madness/mental disease, evil eye, nightmare, impotency)	298	19	0.9
(2) Gastrointestinal (tongue disease, throat (esophagus) disease, tonsillitis, abdominal illness (vomiting, abdominal impelling/bloating), tapeworm, constipation, toothache, ascaris, intestinal diseases, liver disease/jaundice, hemorrhoids, diarrhea, abdominal burning/gastritis, poisonings)	365	41	0.9
(3) Miscellaneous diseases (snake bite, febrile illness, spider bites, swelling)	197	19	0.9
(4) Dermatological system diseases (wound, eczema, Tinea capitis, Tinea corporis, Tinea nigra, scabies/rashes, dandruff, skin peeling, TB (head wound), swelling, warts, gland removal)	225	41	0.8
(5) Infective diseases (malaria, rabies)	33	6	0.8
(6) Musculoskeletal system diseases (rheumatism, backache, stabbing pain)	19	6	0.7
(7) Sensorial diseases (eye disease, night blindness, deafness, trachoma, ear pest, tongue disease, epistaxis, earache)	48	14	0.7
(8) Circulatory system diseases (blood pressure)	9	6	0.4
(9) Metabolism diseases (diabetes)	4	3	0.3
(10) Respiratory system diseases (coughs, asthma, common cold, lung disease)	19	15	0.2
(11) Reproductive system diseases (syphilis, gonorrhea, neonatal death, breast swelling, penis swelling, urinary retention)	8	7	0.1

**Table 3:** Fidelity levels (FL) of 14 most cited traditional medicinal plants.

List of medicinal plants	Family	Therapeutic uses	Ip	Iu	(FL) (%)
*Dodonea angustifolia* L.	Sapindaceae	Malaria	3	3	100
*Dovyalis abyssinica* (A. Rich.) Warb.	Flacourtiaceae	Asthma	4	4	100
*Hagenia abyssinica* (Bruce) J.F. Gmelin	Rosaceae	Tapeworm	53	53	100
*Nigella sativa* L.	Ranunculaceae	Cough	3	3	100
*Urtica simensis* Steudel	Urticaceae	Stomach ulcer	2	2	100
*Rumex abyssinicus* Jacq.	Polygonaceae	Diabetes	3	4	75
*Croton macrostachyus* Del.	Euphorbiaceae	Blood pressure	6	9	66.7
*Rumex nervosus* Vahl	Polygonaceae	Common cold	4	8	50
*Calpurnia aurea* (Ait.) Benth.	Fabaceae	Snake bite	4	13	30.8
*Euphorbia abyssinica* Gmel.	Euphorbiaceae	Rabies	3	30	30
*Kanahia laniflora* (Forssk.) R. Br.	Asclepiadaceae	Scabies	5	27	18.5
*Osyris quadripartita* Decn.	Santalaceae	Trachoma	2	21	9.5

**Table 4:** Preference ranking exercise of 7 species reported to treat blood pressure.

Medicinal plants for blood pressure	Informants labeled A to P																Score	Rank
	A	B	C	D	E	F	G	H	I	J	K	L	M	N	O	P		
*Croton macrostachyus* Del.	6	7		4	5	6	1	1	4	6	2	4	7	2	2	3	60	5th
*D. abyssinica* (A. Rich.) Warb.	1	1	5	6	3	1	5	5	3	1	4	6	6	4	1	4	56	6th
*Lupinus albus* L.	5	5	6	1	7	5	6	3	5	5	3	1	1	7	5	1	66	3rd
*Phragmanthera regularis*	3	3	2	5	4	4	2	2	6	3	6	5	5	6	3	5	64	4th
*Rosa abyssinica* Lindley.	2	2	7	3	2	7	3	4	2	2	1	2	3	1	4	2	47	7th
*Thymus schimperi* Ron.	4	4		2	6	3	4	7	7	4	5	3	2	5	7	7	70	2nd
*Verbascum sinaiticum* Benth.	7	6		4	1	2	7	6	1	7	7	7	4	3	6	6	74	1st

**Table 5:** Preference ranking of 4 medicinal plants reported to treat asthma.

Medicinal plants for asthma	Informants labeled A to P																Score	Rank
	A	B	C	D	E	F	G	H	I	J	K	L	M	N	O	P		
*Verbascum sinaiticum* Benth.	4	4	1	4	2	3	1	1	4	3	3	4	4	2	2	3	45	1st
*Rubia cordifolia* L.	1	1	4	2	1	1	3	4	1	1	4	3	2	3	1	4	36	3rd
*Dovyalis abyssinica* (A. Rich.) Warb.	2	2	3	1	4	2	4	3	2	2	2	1	1	1	4	1	35	4th
*Catha edulis* (Vahl) Forssk. ex Endl.	3	3	2	3	3	4	2	2	3	4	1	2	3	4	3	2	44	2nd

Note: scores in the table indicate ranks given to medicinal plants based on their efficacy.

**Table 6:** Average DMR score of six key informants for 5 medicinal plants with additional uses.

UD	Traditional medicinal plants																														Score	Rank
	*C. spinarum*						*C. macrostachyus*						*D. abyssinica*						*O. europaea*						*R. abyssinica*							
	Informant (I) (1-6)						I						I						I						I							
AT	2	1	2	1	0	3	4	5	5	4	4	5	2	1	0	0	1	0	3	2	2	3	2	2	0	1	1	1	2	1	60	4th
FW	3	2	1	3	4	5	3	1	4	1	3	1	3	4	3	5	2	1	4	5	2	3	4	4	2	4	5	3	2	4	91	2nd
F	5	4	3	5	5	2	0	0	0	0	0	0	4	3	2	2	4	2	2	1	2	3	1	2	5	5	4	3	2	2	73	5th
M	5	5	5	5	4	4	3	3	2	1	4	5	1	2	3	3	4	1	5	4	3	3	4	2	2	3	4	4	5	3	102	1st
CO	1	1	0	1	1	1	1	1	2	3	4	3	2	1	3	2	1	2	5	4	5	4	4	5	2	1	0	1	2	2	65	3rd
GT	84						72						64						95						76							
Rank	2nd						4th						5th						1st						3rd							

Abbreviations: UD = use diversity; AT = agricultural tools; FW = firewood; F = food; M = medicine; CO = construction; GT = grand total.

**Table 7:** Use values of most cited medicinal plants listed by 16 key informants (I).

Medicinal plants	Number of uses (Ui)																∑Ui	*n*	UV
	1	2	3	4	5	6	7	8	9	10	11	12	13	14	15	16			
*Allium sativum* L.	5	2	6	3	4	1	4	6	5	4	3	5	2	1	3	6	60	16	3.8
*Carissa spinarum* L.	3	7	8	5	4	2	5	3	3	4	4	3	8	2	7	7	75	16	4.7
*Catha edulis*	3	4	3	4	1	4	4	2	1	3	2	4	3	4	4	4	50	16	3.1
*Croton macrostachyus*	7	2	5	3	3	7	3	3	4	4	7	3	4	7	5	3	70	16	4.4
*Cucumis ficifolius*	5	3	4	1	4	4	3	4	5	3	4	3	5	3	5	5	61	16	3.8
*C. coeruleum*	3	4	3	3	4	3	4	3	4	1	4	4	4	2	4	3	53	16	3.3
*Echinops kebericho*	5	6	2	5	3	3	6	2	5	3	6	6	3	6	3	5	69	16	4.3
*Embelia schimperi*	4	5	4	2	5	3	5	4	2	5	3	5	4	2	5	2	60	16	3.8

**Table 8:** Medicinal use values of medicinal plants.

Scientific name	No. of informants citing the species	Number of medicinal uses for diseases	Total no. of remedial uses	UVmed
*Rumex nepalensis*	2	7	7	3.5
*Lepidium sativum*	4	3	3	0.75
*Kanahia laniflora*	5	3	3	0.6
*S. longepedunculata*	2	1	1	0.5
*Zehneria scabra*	22	3	3	0.1
*Kalanchoe petitiana*	15	1	1	0.07
*Cucumis ficifolius*	135	6	6	0.04

Note: UVmed = medicinal use values.

**Table 9:** Statistical test of significance, *t*-test, on average number of reported medicinal plants among different informant groups in the Yilmana Densa and Quarit districts.

Parameters	Informant groups	*N*	Average ± SD	*t* value^∗∗^	*p* value^∗^
Age groups	Adults (≤50 years)	207	5.0242 ± 4.05956	7.123	0.008
	Elders (>50 years)	188	6.2606 ± 5.12738		
Gender	Females	129	3.9070 ± 0.91398	17.196	0
	Males	266	5.7970 ± 0.76031		
Informant categories	General informants	299	4.8087 ± 0.84817	40.147	0
	Key informants	96	8.0825 ± 0.84464		
Level of education	Illiterates	290	5.3241 ± 0.46731	4.264	0.04
	Literates	105	6.4095 ± 0.00326		

^∗^Significant difference (*p* < 0.05); ^∗∗^*t* (0.05) (two tailed), df = 393, *N* = number of respondents.

**Table 10:** Ranking of the threats for loss of traditional knowledge.

Threats of for the loss of TK	Informants labeled A to O															Score	Rank
	A	B	C	D	E	F	G	H	I	J	K	L	M	N	O		
The dwindling of traditional schools'/churches' students in number	3	1	2	3	2	3	4	1	2	3	1	4	2	3	2	36	3rd
The expansion of modern pharmacies	5	5	5	5	5	5	5	5	5	5	5	5	5	5	5	75	1st
The expansion of modern education	4	2	4	4	3	4	3	4	3	4	4	3	3	4	4	53	2nd
The migration of healers to towns or cities	2	3	3	2	1	1	1	2	1	1	2	2	4	1	1	27	5th
Scarcity of medicinal plants	1	4	1	1	4	2	2	3	4	2	3	1	1	2	3	34	4th

Note: the highest value was 5 while the least value was 1. The highest value (5) was given by informants for the causes considered to be the greatest threat.

## Data Availability

All data sets are included within the article.
